# Macrophage autophagy regulates mitochondria‐mediated apoptosis and inhibits necrotic core formation in vulnerable plaques

**DOI:** 10.1111/jcmm.14715

**Published:** 2019-10-29

**Authors:** Qingqing Xiao, Xinyu Che, Bin Cai, Zhenyu Tao, Hengyuan Zhang, Qin Shao, Jun Pu

**Affiliations:** ^1^ Department of Cardiology Renji Hospital Shanghai Jiao Tong University School of Medicine Shanghai China; ^2^ Shanghai Institute of Rheumatology Renji Hospital Shanghai Jiao Tong University School of Medicine Shanghai China

**Keywords:** apoptosis, autophagy, macrophage, mitochondrial dysfunction, vulnerable plaque

## Abstract

The vulnerable plaque is a key distinguishing feature of atherosclerotic lesions that can cause acute atherothrombotic vascular disease. This study was designed to explore the effect of autophagy on mitochondria‐mediated macrophage apoptosis and vulnerable plaques. Here, we generated the mouse model of vulnerable carotid plaque in ApoE^−/−^ mice. Application of ApoE^−/−^ mice with rapamycin (an autophagy inducer) inhibited necrotic core formation in vulnerable plaques by decreasing macrophage apoptosis. However, 3‐methyladenine (an autophagy inhibitor) promoted plaque vulnerability through deteriorating these indexes. To further explore the mechanism of autophagy on macrophage apoptosis, we used macrophage apoptosis model in vitro and found that 7‐ketocholesterol (7‐KC, one of the primary oxysterols in oxLDL) caused macrophage apoptosis with concomitant impairment of mitochondria, characterized by the impairment of mitochondrial ultrastructure, cytochrome c release, mitochondrial potential dissipation, mitochondrial fragmentation, excessive ROS generation and both caspase‐9 and caspase‐3 activation. Interestingly, such mitochondrial apoptotic responses were ameliorated by autophagy activator, but exacerbated by autophagy inhibitor. Finally, we found that MAPK‐NF‐κB signalling pathway was involved in autophagy modulation of 7‐KC–induced macrophage apoptosis. So, we provide strong evidence for the potential therapeutic benefit of macrophage autophagy in regulating mitochondria‐mediated apoptosis and inhibiting necrotic core formation in vulnerable plaques.

## 1 INTRODUCTION

Atherosclerosis is a chronic lipid‐driven inflammatory disease that can cause life‐threatening complications, such as acute myocardial infarct, stroke and aortic aneurysm.^1^ Vulnerable plaques are more prone to rapid plaque progression which is more likely to trigger acute obstructive vascular disease. Vulnerable plaques have a typical morphology, namely a thin, inflamed fibrous cap covering a large and soft lipid‐rich necrotic core.^2^ Moreover, larger necrotic core (ie the intraplaque macrophage apoptosis) is associated with an vulnerable plaque phenotype, which is more likely to rupture and manifest as acute clinical complications.^3^, ^4^ Macrophage apoptosis, a crucial feature of unstable plaque lesions, has been recognized as a critical step in the formation of the necrotic core.^5^ Recent studies have demonstrated that regulation of macrophage apoptosis is the key to stabilize unstable plaques and modulate the progression of atherosclerosis.^6^, ^7^ Therefore, elucidating the regulatory mechanisms of necrotic core involving in macrophage apoptosis may yield novel targets in vulnerable plaque treatment and in crucial applications to vascular biology and clinical medicine.

Autophagy is a highly evolutionarily conserved process to recycle cytosolic materials (including proteins and organelles) and maintain intracellular homeostasis.^8^ During this process, cytosolic materials are sequestrated into autophagosome and degraded by fusing with lysosomes. Growing evidence reveals that basal autophagy has crucial role in anti‐atherosclerosis during early stages.^9^, ^10^ In advanced atherosclerotic plaques, autophagy becomes defective with over‐exposing atherosclerotic factors, such as 7‐ketocholesterol^11^ and oxLDL.^12^ Previous studies have demonstrated that basic autophagy deficiency by specific Atg5 knockout could accelerate atherosclerotic.^13^ Moreover, our previous studies have suggested that up‐regulated autophagy level by atorvastatin could inhibit inflammatory cytokines secretion and lipid accumulation and even suppress atherosclerosis progression both in vitro and in vivo.^14^, ^15^ However, the beneficial effects of autophagy on vulnerable plaques and the potential mechanism are still little known.

Mitochondrial dysfunction participates in a series of diseases,^16^, ^17^ such as acute renal injury,^18^ neurodegeneration,^19^ myocardial ischaemia/reperfusion injury^20^ and heart failure.^21^ Increased opening of mitochondrial permeability transition pore (MPTP) results in mitochondrial dysfunction, which includes mitochondrial membrane potential (MMP) loss, cytochrome c loss from the intermembrane space, excessive reactive oxygen species (ROS) generation and mitochondrial fission.^22^ Recent data suggest that mitochondrial dysfunction plays a crucial role in macrophage death in advanced lesion.^23^, ^24^ Also, cholesterol loading of mouse peritoneal macrophages induces cells apoptosis by damaging mitochondria.^25^ Autophagy is the main way to degrade impaired mitochondria. A previous study has testified that autophagy plays a protective role in NBT‐induced MCF‐7 cells apoptosis and mitochondrial dysfunction.^26^ Thus, we guess that accumulation of damaged mitochondria will lead to macrophage apoptosis, favouring plaque rupture in vulnerable plaques, which can be ameliorated by autophagy.

Here, we reported our findings that macrophage autophagy could inhibit necrotic core formation and development in vulnerable plaques. Meanwhile, macrophage autophagy could regulate macrophage apoptosis both in vivo and in vitro. Importantly, we demonstrated for the first time in macrophages that autophagy could effectively ameliorate mitochondrial dysfunction mainly via regulating the mitochondrial membrane potential, rescuing the damage of mitochondrial ultrastructure, inhibiting cytochrome c release and mitigating excessive ROS (reactive oxygen species) release. Our findings may suggest the vasoprotection of autophagy against development of vulnerable plaques and provide key implications for the efficacy of autophagy‐targeted treatments for vulnerable plaques.

## 2 MATERIALS AND METHODS

### 2.1 Materials

See the Supplementary Methods for details.

### 2.2 Animals and surgical protocol

All animal experiments were carried out in accordance with guidelines approved by the Shanghai Jiaotong University school of Medicine Institutional Animal Care and Use Committee. Eight‐week‐old male ApoE^−/−^C57BL/6 mice were purchased from Nanjing Biomedical Research Institute of Nanjing University (Nanjing, China). As previously described,^27^, ^28^ ligation surgery was implemented with a dissecting microscope, which could achieve the combined partial ligation of the left common carotid artery (LCCA) and left renal artery. Briefly, mice were anesthetized and maintained on a heating panel at 37°C. Left common carotid artery (LCCA) and all its branches were exposed by blunt dissection. Then, all branches of LCCA, except for the left thyroid artery, were ligated to reduce flow in the LCCA. The partial left renal artery surgery was carried out 1 week after ligation of partial LCCA as described previously. Mice were anesthetized, and the left kidney was exposed through an abdominal incision. The left renal artery was ligated along with a pin gauge (outer diameter = 0.12 mm) after isolation by blunt dissection. Subsequently, the pin gauge was taken out to leave a tight artery. The pin gauge was commercially available (Jining Hengsheng Precise Measuring and Cutting Co Ltd), and its size was verified using a micrometer caliper.

Then, the mice were divided into three groups: control (saline, n = 15), rapamycin (30 mg/kg/d, n = 15) and 3‐methyladenine (20 mg/kg/d, n = 15). Both rapamycin and 3‐MA were dissolved in saline, and mice were administered intragastrically for 8 weeks after combined surgery.

### 2.3 MRI

Details are in the Supplementary Methods.

### 2.4 Tissue collection and analysis

At postoperative week 8, mice were killed with deep anaesthesia for blood collection. Then, the mice were fixed by perfusion with saline, and specimens of carotid arteries were obtained and filled with optical cutting temperature (OCT) compound (Tissue‐TEK) or paraffin. Serially sectioned frozen carotid artery specimens (7 μm) were stained with haematoxylin and eosin (H&E, Sigma), Masson's trichrome (Sigma) and Oil Red O (Sigma). In haematoxylin and eosin staining, intraplaque haemorrhage is defined as the presence of the deposition of erythrocytes, and/or fibrin clots inside the plaque and plaque rupture are defined as discontinuous fibrous cap.

### 2.5 Cell culture

RAW264.7 cells (murine macrophage cell line) from the Institute of Biochemistry and Cell Biochemistry and Cell Biology (Shanghai, China) were cultured in Dulbecco's Modified Eagle's Medium (DMEM) supplemented with 10% foetal bovine serum at 37°C in a humidified 5% CO_2_ atmosphere. For pharmacological treatment, macrophage cells were cocultured with Rap (5 μmol/L), 3‐MA (3 mmol/L) or CQ (25 μmol/L) for 1 hour and subsequently incubated with 60 μmol/L 7‐KC for 24 hours to induce foam cells and apoptotic cells before being harvested.

### 2.6 Western blot analysis

Details are described in the Supplementary Methods.

### 2.7 Flow cytometric analysis of apoptosis by annexin V/PI staining

Details are described in the Supplementary Methods.

### 2.8 Real‐time quantitative PCR

Details are described in the Supplementary Methods.

### 2.9 Macrophage transfection with siRNA

Details are described in the Supplementary Methods.

### 2.10 Immunofluorescence staining

Details are in the Supplementary Methods.

### 2.11 Terminal deoxynucleotidyl transferase dUTP nick end labelling (TUNEL) assay

Details are in the Supplementary Methods.

### 2.12 Transmission electron microscopy (TEM)

Details are in the Supplementary Methods.

### 2.13 Detection of intracellular ROS

Details are in the Supplementary Methods.

### 2.14 Measurement of mitochondrial membrane potential (ΔΨm)

Details are in the Supplementary Methods.

### 2.15 Statistics

Results were presented as mean ± SEM from at least three separate experiments and conducted using GraphPad Prism 6 software (GraphPad). Two experimental groups were analysed by the two‐tailed paired Student's *t* test. Three or more groups were assessed by ANOVA with a Newman‐Keuls test. *P* values <.05 were considered statistically significant.

## 3 RESULTS

### 3.1 Autophagy activation inhibited vulnerable plaque progression in ApoE^−/−^ mice

**Figure 1:**
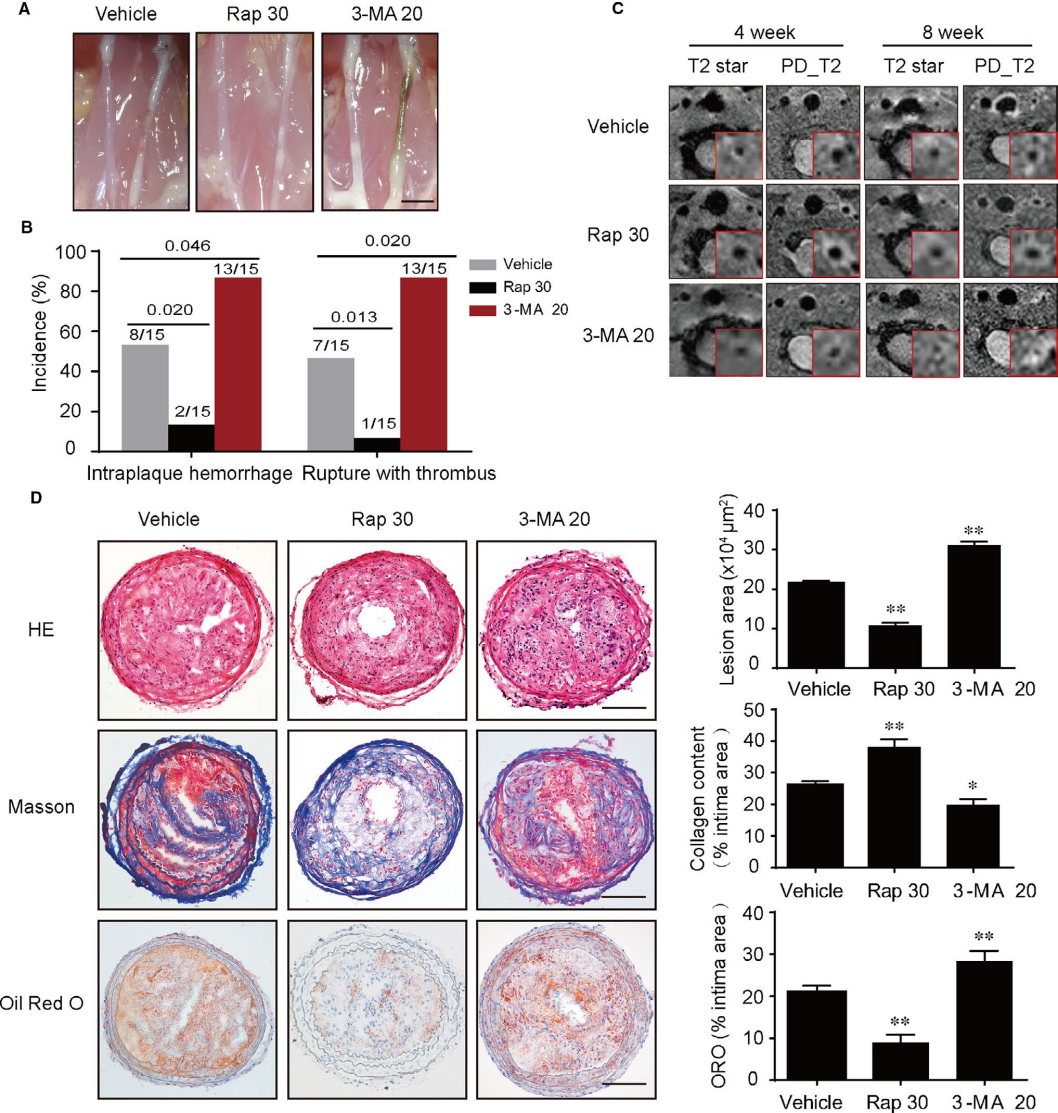
Autophagy activation induced by rapamycin inhibits vulnerable plaque progression in ApoE^−/−^ mice. A, Anatomical view of common carotid artery from ApoE^−/−^ mice treated with vehicle, Rap 30 and 3‐MA 20 under a dissecting microscope, scale bar = 2.5 mm. B, Incidence of intraplaque haemorrhage and rupture with thrombus (left). C, Representative T2 stat and PD T2 magnetic resonance (MR) images of mice common carotid artery, collected at 4 and 8 wk after surgery. The insets in each scan were cropped, and the left common carotid artery was enlarged to highlight in the red frame. D, Representative images of left common carotid artery cross sections stained with haematoxylin and eosin (H&E), Masson's trichrome and Oil Red O from different groups, quantification of lesion area of carotid arteries, ORO^+^ and collagen^+^ areas relative to total lesion area, scale bar = 100 μm. Data were presented as mean ± SEM of at least three independent experiments. *P < .05 compared with vehicle group, **P < .01 compared with vehicle group. Vehicle, mice were administrated with saline solution alone. Rap 30, rapamycin at 30 mg/kg/d; 3‐MA 20, 3‐methyladenine at 20 mg/kg/d

To investigate the effects of autophagy on vulnerable plaque progression, ApoE^−/−^ mice were carried out an animal model for vulnerable plaques (Figure S1A‐C). Also, rapamycin activated macrophage autophagy, as demonstrated by increased ratio of LC3II/LC3I and decreased level of SQSTM1/p62 in aortic plaques, whereas 3‐MA manifested defective autophagy (Figure S2A). Furthermore, fluorescence microscopic images demonstrated that rapamycin significantly increased the extent of positive staining for LC3B and decreased the extent of positive p62 staining in vulnerable plaques. Nevertheless, 3‐MA inhibited macrophage autophagy in plaques (Figure S2B,C). Next, we found that activation of autophagy by rapamycin reduced the incidence of intraplaque haemorrhage (2/15 vs 8/15, *P* = .020) and of rupture with thrombus (1/15 vs 7/15, *P* = .013), whereas the inhibition of autophagy by 3‐MA increased incidence of intraplaque haemorrhage (13/15 vs 8/15, *P* = .046) and of spontaneous plaques rupture with thrombus (13/15 vs 7/15, *P* = .020; Figure 1A,B). Magnetic resonance imaging of the LCCA section revealed that the differences between the plaque size and the residual luminal area were not significant in all groups of ApoE^−/−^mice at 4 weeks after ligation. However, the rapamycin could significantly decrease the plaque area and increase the residual luminal area at 8 weeks after ligation. In contrast to the vehicle group, 3‐MA could markedly increase these indexes (Figure 1C). Next, we determined the effects of autophagy on vulnerable plaque morphology and components. Importantly, we found that rapamycin could significantly decrease the plaque area and lipid deposition, while increase the amount of collagen in the vulnerable plaques. On the contrast, 3‐MA was able to reverse these trends (Figure 1D). Moreover, no significant differences were observed in systolic pressure, diastolic pressure and mean arterial blood pressure (MABP) in these three groups (Figure 1D‐F). Thus, these data suggested that activation of autophagy could improve stability of vulnerable plaque.

### 3.2 Autophagy inhibited the formation of plaque necrosis via suppressing macrophage apoptosis in vulnerable plaques

**Figure 2:**
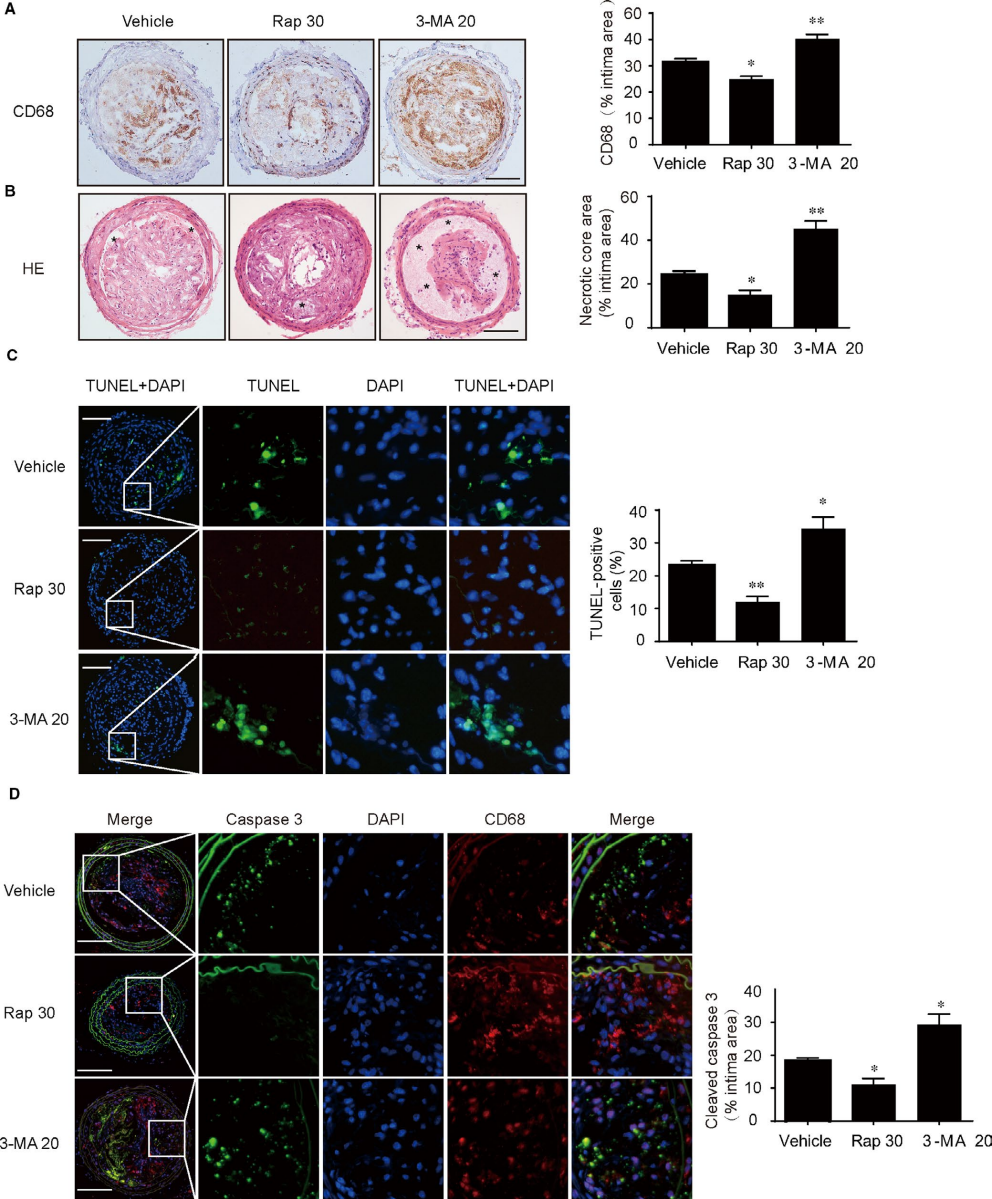
Effects of autophagy on macrophage apoptosis and plaque necrosis. A, Immunohistochemical staining of left common carotid artery tissue with CD68 from vehicle‐treated, rapamycin‐treated and 3‐MA‐treated mice, quantification of CD68^+^ areas relative to total lesion area, scale bar = 100 μm. B, Representative images of carotid artery cross sections stained with haematoxylin and eosin (H&E) from ApoE^−/−^ mice (*, necrotic areas), quantification of the necrotic core area relative to total lesion area, scale bar = 100 μm. C, Representative images of apoptotic cells in lesions by TUNEL assay, the number of TUNEL‐positive cells was measured and quantitated, scale bar = 100 μm. D, Representative images of carotid artery cross sections were stained with specific antibodies against the macrophage marker (CD68) and co‐probed with antibodies against the marker of macrophage apoptosis (cleaved caspase‐3), scale bar = 100 μm. Data were presented as mean ± SEM of at least three independent experiments. *P < .05 compared with vehicle group, **P < .01 compared with vehicle group. Vehicle, mice were administrated with saline solution alone. Rap 30, rapamycin at 30 mg/kg/d; 3‐MA 20, 3‐methyladenine at 20 mg/kg/d

The apoptosis of macrophages promotes the formation of necrotic core in plaques and thereby increases the vulnerability of plaques.^29^ Next, we determined the effects of autophagy on the macrophage apoptosis and plaque necrosis. As shown in Figure 2A, we observed that the Rap group exhibited significantly smaller CD68‐positive areas than the vehicle group, whereas these effects were abolished by 3‐MA. These data revealed that autophagy could reduce macrophages infiltration. Furthermore, we found a significant decrease in plaque necrosis in lesions with rapamycin, whereas the 3‐MA group showed the opposite (Figure 2B). Thus, the increase in plaque necrosis was associated with high rates of macrophages infiltration. Besides, there was a significant reduction in the percentage of cells with TUNEL‐positive cells in the lesions of Rap‐treated mice compared with the control group (Figure 2C), whereas 3‐MA group reversed these indexes. Moreover, we found that CD68^+^ macrophages in carotid plaque lesions from rapamycin‐treated ApoE^−/−^ mice exhibited decreased expression of cleaved caspase‐3, indicating that autophagy could suppress macrophage apoptosis in vulnerable plaques in vivo (Figure 2D). Taken together, these results demonstrated that activation of autophagy in mice could inhibit the formation of plaque necrosis via suppressing macrophage apoptosis in vulnerable plaques.

### 3.3 7‐ketocholesterol induced apoptosis in RAW264.7 cells

We further investigated the detailed mechanism of autophagy on macrophage apoptosis and the stability of vulnerable plaques in vitro. 7‐ketocholesterol (7‐KC) (Figure S3A), one of the primary oxysterols in oxLDL, plays a pivotal role in macrophage apoptosis.^30^ To examine the effects of 7‐KC on the viability of RAW264.7 cells, cells were treated with various concentrations (0, 10, 20, 40, 60, 80 μmol/L) for 24 hours. Dose‐dependent manner was observed with 7‐KC treatment (Figure S3B). Based on the results of MTT, cells were treated with 60 μmol/L 7‐KC for different time (0, 3, 6, 12, 18 and 24 hours). As shown in Figure S3C, 7‐KC inhibited cell viability in a time‐dependent manner. To further determine macrophage apoptosis in response to 7‐KC treatment, caspase‐3 activity, caspase‐9 activity (mitochondrial‐mediated cytochrome c‐dependent protein) and PARP levels were assessed at different concentrations and time‐points as above. Activity of caspase‐3 and caspase‐9 was dose‐ and time‐dependent in the 7‐KC–treated groups. Meanwhile, the activity of PARP showed similar change (Figure S3D,E). These results demonstrated that 7‐KC–induced cell apoptosis in a dose‐ and time‐dependent manner was mainly associated with a mitochondrial‐mediated caspase cascades activation pathway.

### 3.4 Autophagy flux was blocked during 7‐KC–induced macrophages apoptosis

To evaluated the effects of 7‐KC on autophagy flux in macrophages, cells were treated with 60 μmol/L 7‐KC at various time intervals. We found that accumulation of SQSTM1/P62 was paralleled to an increase in the ratio of LC3II/LC3I, reflecting impairment in the autophagy flux (Figure S4A). This effect was similar to the blockade of the autophagy flux induced by chloroquine (CQ; Figure S4C). In the meanwhile, rapamycin had no influence on the ratio of LC3II/LC3I, but decreased accumulation of SQSTM1/P62 as compared with cells treated with 7‐KC alone, indicating a recovery of impaired autophagy (Figure S4B). Moreover, 3‐MA decreased the ratio of LC3II/LC3I and showed no obvious changes in the SQSTM1/P62 abundance as compared with cells treated with 7‐KC alone (Figure S4C), indicating non‐improved autophagic flux, which was consistent with immunofluorescence staining (Figure S4D). Taken together, these data supported that rapamycin could restore the impaired autophagy induced by 7‐KC. However, 3‐MA or CQ may block the autophagy flux.

### 3.5 Enhancement of autophagy by rapamycin attenuated 7‐KC–induced macrophage apoptosis

**Figure 3:**
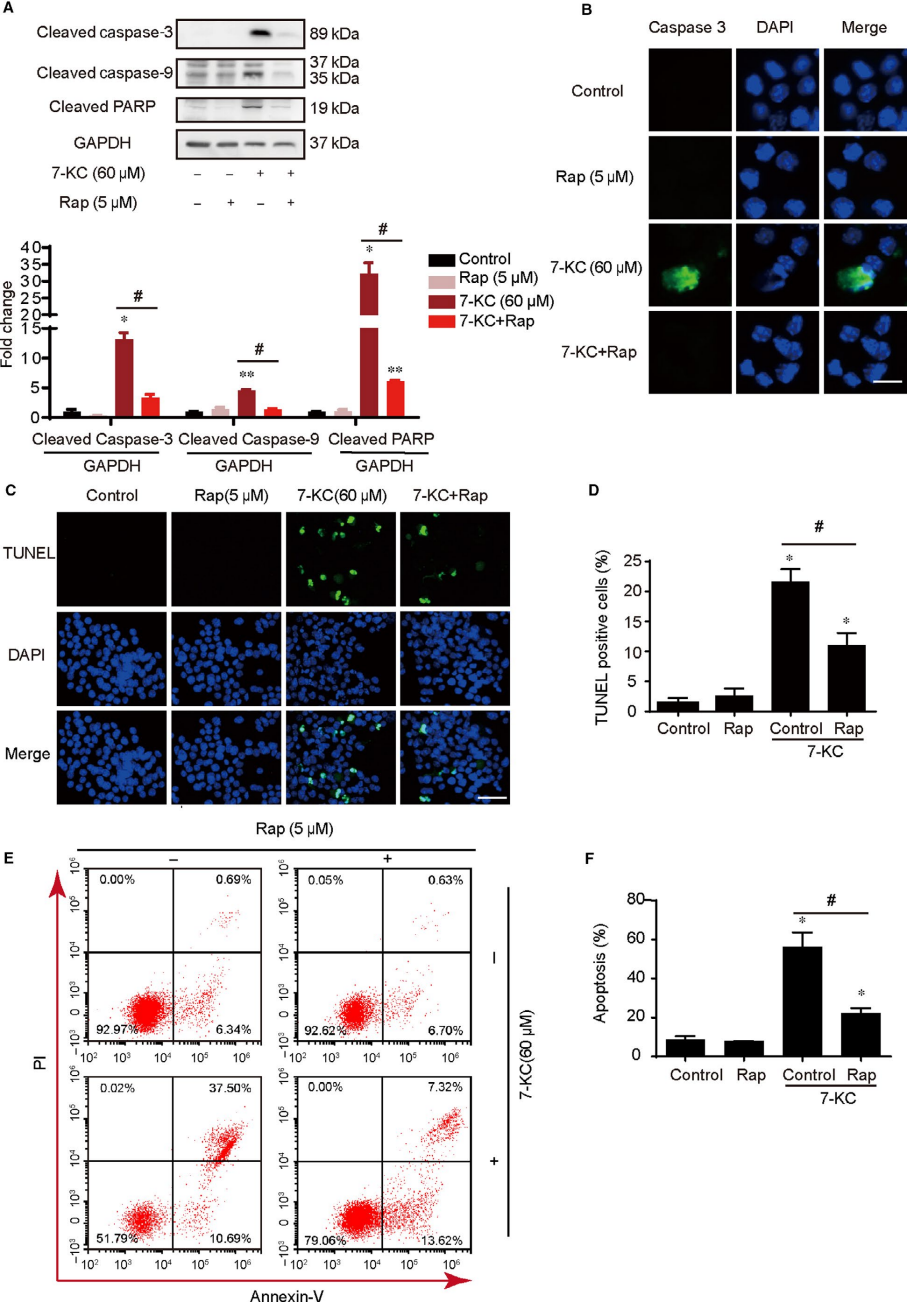
Enhancement of autophagy by rapamycin attenuated cells apoptosis induced by 7‐KC in RAW264.7 cells. RAW264.7 cells were treated with Rap (5 μmol/L) for 60 min and then exposed to 7‐KC (60 μmol/L) for additional 24 h. A, Western blot analysis of cleaved caspase‐3, cleaved caspase‐9 and cleaved PARP expression in the whole cells. B, Cells were stained by cleaved caspase‐3 (green) and DAPI (blue) and analysed by fluorescence microscopy, scale bar = 15 μm. C, D, Representative images of TUNEL staining of macrophages showed the apoptotic cells (apoptotic cells stained in green and nucleus stained in blue with DAPI). The number of TUNEL‐positive cells was measured and quantitated. Scale bar = 50 μm. E, F, RAW 264.7 cells were cotreated with 60 μmol/L 7‐KC and 5 μmol/L Rap for 24 h compared with control or 7‐KC and Rap treatment alone, and apoptosis was examined by using annexin V with PI staining with flow cytometry. Data were presented as mean ± SEM of at least three independent experiments. *P < .05 vs control, ** *P* < .01 vs control, #*P* < .05 vs 7‐KC group

As shown in Figure 3A, pretreatment with Rap (rapamycin, 5 μmol/L) prevented 7‐KC–induced RAW264.7 cells apoptosis, which was shown in the down‐regulated expression of cleaved caspase‐3, caspase‐9 and cleaved PARP. Furthermore, fluorescence microscopic images demonstrated that Rap inhibited the intracellular expressions of cleaved caspase‐3 (Figure 3B), which was in conformity to the results of Western blot. Next, this finding was further supported via TUNEL staining, which exhibited a remarkable reduction in the percentage of TUNEL‐positive cells in the pretreatment of rapamycin (Figure 3C,D). Meanwhile, the cotreatment of 7‐KC and rapamycin for 24 hours in RAW264.7 cells resulted in a marked decrease in apoptosis according to the annexin V/propidium iodide (PI) staining test (Figure 3E,F). Next, we used primary macrophages of murine to verify that rapamycin did inhibit macrophage apoptosis. Similarly, we examined the expression of apoptosis‐associated proteins stimulated by 7‐KC in the absence or presence of rapamycin as above. As shown in Figure S5, 7‐KC could obviously enhance the proteins of cleaved caspase‐3 and cleaved PARP, as well as the fluorescence intensity of cleaved caspase‐3, and the percentage of TUNEL‐positive cells compared with the control in murine macrophages, whereas applying rapamycin could effectively reverse these trends. Taken together, these data suggested that activation of autophagy protected against macrophage apoptosis.

### 3.6 Inhibition of autophagy aggravated 7‐KC–induced cells apoptosis in RAW264.7 cells

**Figure 4:**
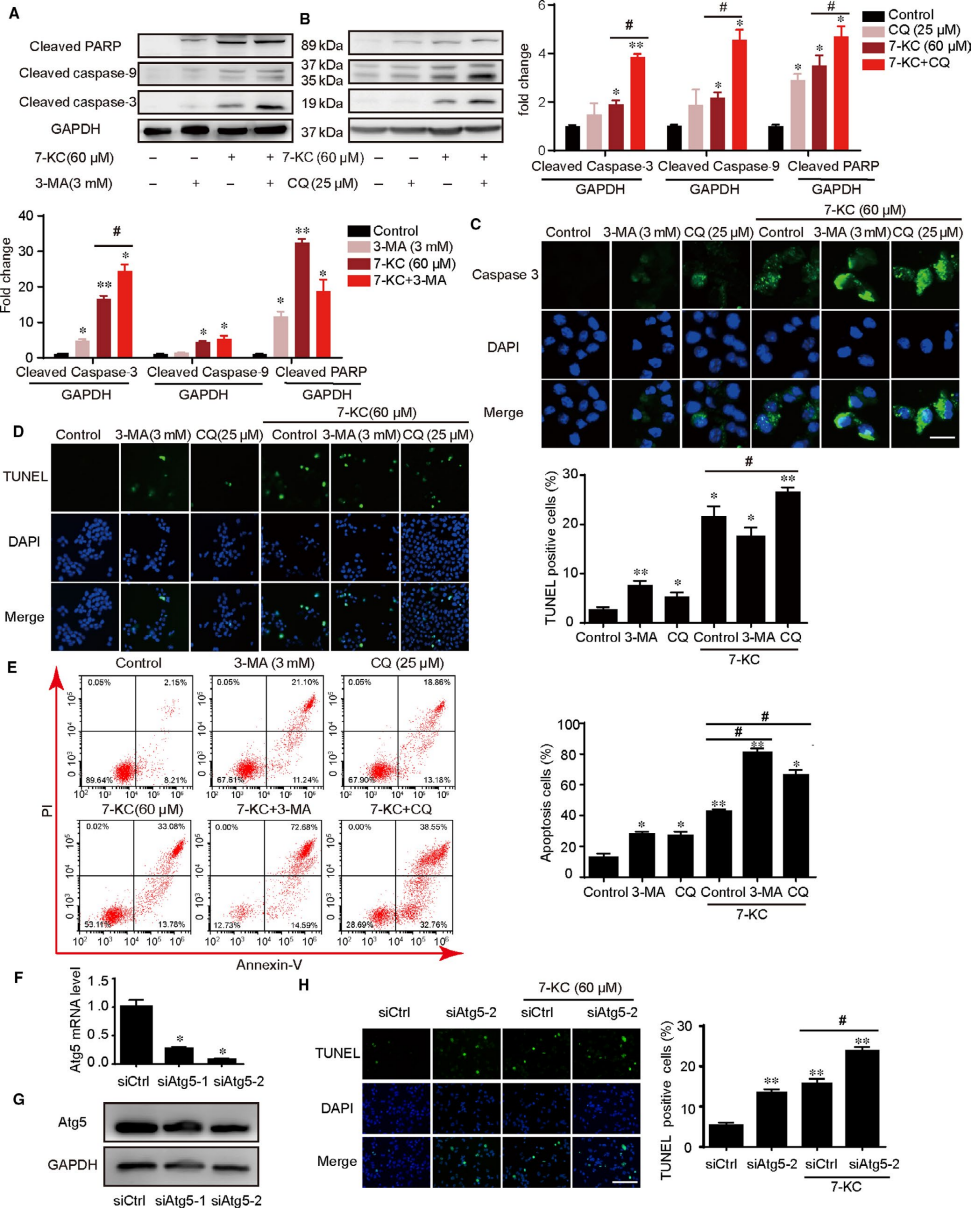
Inhibition of autophagy by 3‐MA or chloroquine aggravated 7‐KC–induced cells apoptosis of RAW264.7 cells. A, RAW264.7 cells were cotreated with 60 μmol/L 7‐KC and 25 μmol/L CQ for 24 h compared with control, 7‐KC or CQ treatment alone, and total cellular extract of cells was prepared and subjected to Western blot by using antibodies against cleaved caspase‐3, cleaved caspase‐9 and cleaved PARP. B, Cells were cotreated with 60 μmol/L 7‐KC and 3 mmol/L 3‐MA for 24 h compared with control, 7‐KC or 3‐MA treatment alone, and total cellular extract of cells was prepared and subjected to Western blot. C, Cells were treated with 7‐KC in the absence or presence of 3‐MA or CQ for 24 h. Then, cells were stained with cleaved caspase‐3 (green) and DAPI (blue) and analysed by fluorescence microscopy, scale bar = 15 μm. D, Representative images of TUNEL staining of macrophages showed the apoptotic cells (apoptotic cells stained in green and nucleus stained in blue with DAPI). The number of TUNEL‐positive cells was measured and quantitated, scale bar = 60 μm. E, Macrophage apoptosis was examined by using annexin V/PI staining with flow cytometry. Data were presented as mean ± SEM of at least three independent experiments. *P < .05 vs control, **P < .01 vs control, #*P* < .05 vs 7‐KC group. F, Atg5 mRNA level was measured by RT‐PCR after transfection with control siRNA(siCtrl) or Atg5 siRNAs(siAtg5). *P < .05 compared with control siRNA group. G, The effect of transfection with Atg5 siRNAs was tested by Western blot. H, Transfection with control siRNA or Atg5 siRNA(siAtg5‐2), subsequently cells were treated with 7‐KC (60 μmol/L) for additional 24 h. Macrophage apoptosis was measured by TUNEL staining, scale bar = 60 μm. Data were presented as mean ± SEM of at least three independent experiments. ** *P* < .01 vs control siRNA, #*P* < .05 vs control siRNA +7‐KC

To further demonstrate the role of blocking autophagy on macrophage apoptosis, we exposed cells to the autophagy inhibitors 3‐methyladenine (3‐MA, 3 mmol/L) or chloroquine (CQ, 25 μmol/L) for 1 hour followed by stimulating with 7‐KC (60 μmol/L) and found higher expression of apoptotic proteins including cleaved caspase‐3, caspase‐9 and cleaved PARP, as Figure 4A,B showed. These data manifested that inhibition of autophagy augmented macrophage apoptosis in response to 7‐KC stimuli. In the meanwhile, the fluorescence intensity of cleaved caspase‐3 was significantly increased in the co‐incubation with 3‐MA or CQ group (Figure 4C). Furthermore, TUNEL staining showed macrophages treated with the inhibitor of autophagy and 7‐KC, alone or concurrently, led to a remarkable increase in apoptosis induction, as shown by the percentage of TUNEL‐positive cells (Figure 4D). Moreover, we found that the cotreatment of 7‐KC and autophagy inhibitors (3‐MA or CQ) for 24 hours in RAW264.7 cells resulted in a marked increase in apoptosis according to the annexin V/propidium iodide (PI) staining test (Figure 4E). Next, we explore autophagy inhibition in macrophages through siRNA‐mediated knockdown of the autophagy‐specific gene. In TUNEL assay, RAW264.7 cells underwent apoptosis process after blocking autophagy with Atg5 siRNA or high‐dose 7‐KC stimulation. Moreover, these macrophages showed an increase in apoptosis, when pretreated with Atg5 siRNA compared with control siRNA (Figure 4F‐H). Thus, inhibition of autophagy through knockdown of Atg5 aggravated 7‐KC–induced cells apoptosis in RAW264.7 cells. Therefore, these results proved that inhibition of autophagy enhanced 7‐KC–induced macrophage apoptosis.

### 3.7 Autophagy prevented macrophage apoptosis by ameliorating mitochondrial dysfunction

**Figure 5:**
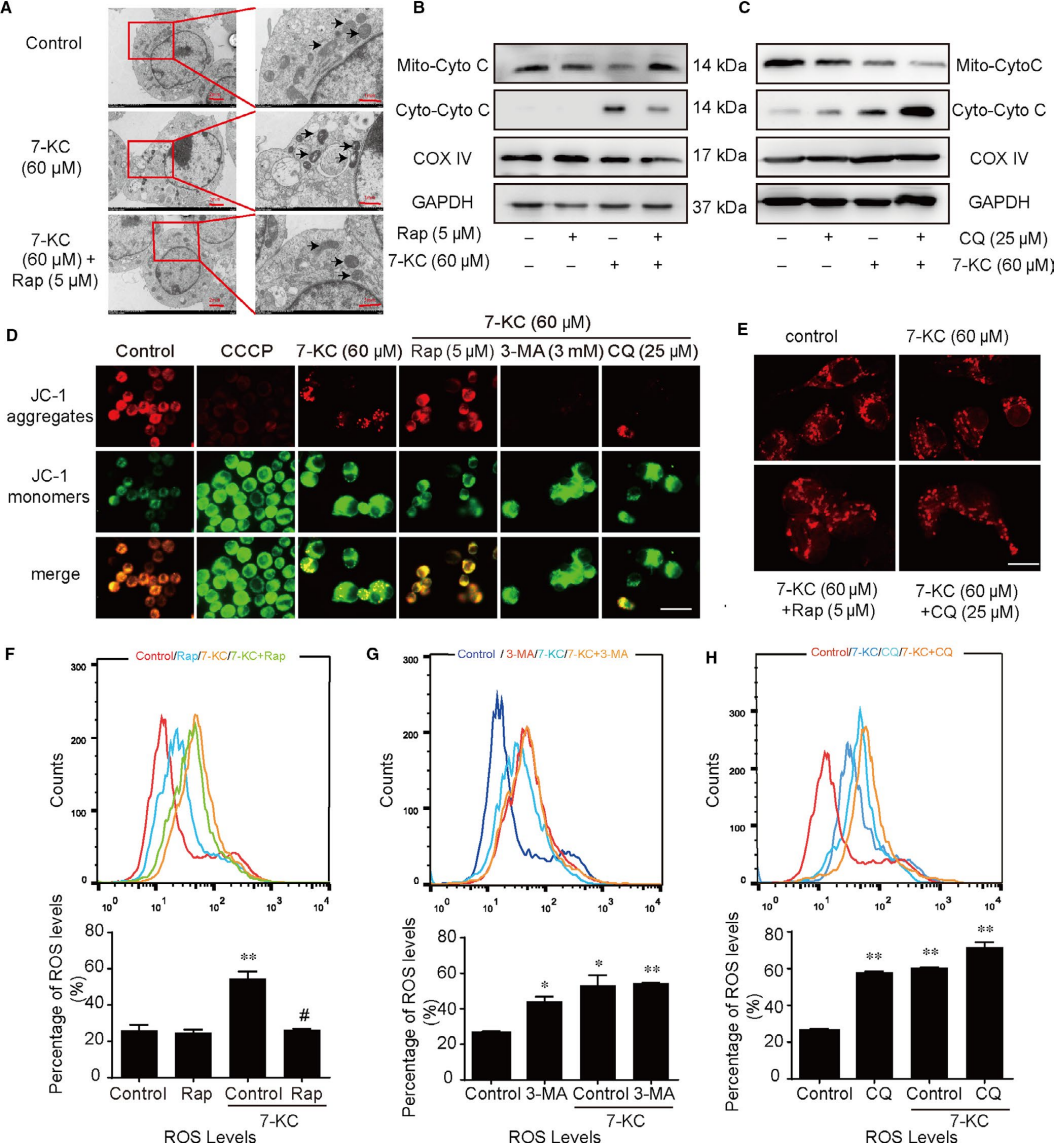
Autophagy prevented RAW264.7 cells apoptosis by ameliorating mitochondrial dysfunction. A, RAW264.7 cells were treated with 7‐KC (60 μmol/L) in the absence or presence of Rap (5 μmol/L) for 24 h. Representative TEM images of RAW264.7 cells. Mitochondria were pointed out by thin arrows. B, RAW264.7 cells were treated with 7‐KC (60 μmol/L) in the absence or presence of Rap (5 μmol/L) for 24 h. Cytochrome c (Cyto C) distribution was analysed by Western blot on mitochondrial and cytosolic fractions. C, RAW264.7 cells were treated with 7‐KC (60 μmol/L) in the absence or presence of CQ (25 μmol/L) for 24 h. Cytochrome c distribution was analysed by Western blot. GAPDH and COX IV were used as controls for the cytosolic and mitochondria fraction, respectively. D, Representative images of mitochondrial depolarization by JC‐1 staining, scale bar = 25 μm. E, Confocal images of RAW264.7 cells stained with Mito Tracker for mitochondria (red). Typical images represented were from cells treated with 7‐KC or co‐incubation with Rap (5 μmol/L) or CQ (25 μmol/L) for 24 h, scale bar = 15 μm. F‐H, The fluorescent label probe DCFH‐DA was used to detect intracellular ROS production in RAW264.7 cells treated with Rap (5 μmol/L), 3‐MA (3 mmol/L) or CQ (25 μmol/L) by flow cytometry. Data were presented as mean ± SEM of at least three independent experiments. *P < .05 vs control, **P < .01 vs control, #*P* < .05 vs 7‐KC group

Experiments to determine whether autophagy could rescue the 7‐KC–mediated changes of mitochondrial ultrastructure that induced apoptosis in RAW264.7 cells were conducted. As shown in Figure 5A, in the control group, macrophages displayed intact mitochondrial structure characteristics. After treatment with 7‐KC, mitochondria exhibited extensive damage, with swollen, vacuolar and even fractured structures. In the presence of rapamycin(60 μmol/L), the mitochondrial ultrastructural morphology exhibited varying degrees of recovery, with few swollen and slightly broken mitochondria. These results indicated that rapamycin efficiently ameliorated the 7‐KC–induced ultrastructural damage of mitochondria in macrophages.

We next investigated whether autophagy could induce the translocation of cytochrome c. As shown in Figure 5B,C, 7‐KC significantly increased the cytoplasmic cytochrome c and decreased the mitochondrial cytochrome c. Importantly, pretreatment with rapamycin could reduce the level of cytoplasmic cytochrome c released from the mitochondria. On the contrary, pretreatment with CQ markedly increased the level of cytoplasmic cytochrome c. Meanwhile, we found more green fluorescence intensity in the 7‐KC group, which demonstrated the decline of mitochondrial membrane potential (Δψm) in macrophages. Then, pretreatment with rapamycin attenuated the loss of Δψm, but autophagy inhibitor (3‐MA or chloroquine) reversed these effects (Figure 5D). Mitochondrial fission plays a pivotal role in mitochondrial apoptosis, which leads to mitochondrial fragmentation.^31^ As shown in Figure 5E, mitochondrial network of macrophage cells in the control group was interconnected and extensive throughout the cells. Interestingly, the mitochondrial network of cells in the 7‐KC group was shorter and more fragmented. However, combination with CQ did not change the dot‐shaped distribution compared with 7‐KC group. Meanwhile, mitochondria slightly elongated in the co‐incubation with rapamycin group.

Reactive oxygen species is produced by dysfunctional mitochondria which induces cells apoptosis.^32^ We further explored the effect of autophagy on ROS generation in 7‐KC–induced macrophage apoptosis. Figure 5F‐H showed that rapamycin could attenuate 7‐KC–induced ROS production, whereas autophagy inhibitor (3‐MA or CQ) could show a remarkably increased ROS levels.

Therefore, we concluded that autophagy could ameliorate mitochondrial function through reduction of MMP lose, ROS generation, cytochrome c release, mitochondrial fission and damaged mitochondrial structure.

### 3.8 Autophagy inhibited apoptosis by MAPK‐NF‐κB signalling pathway

**Figure 6:**
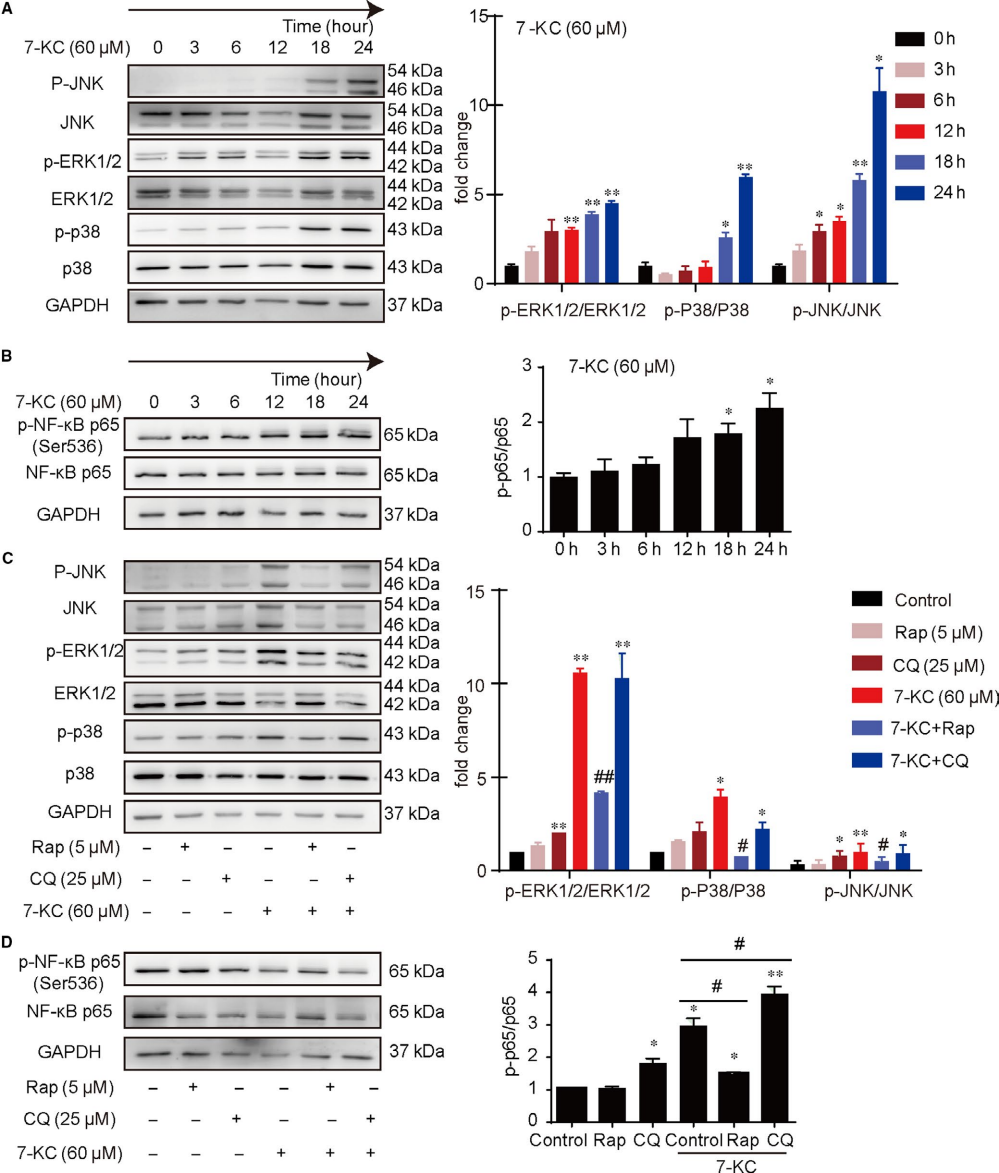
Autophagy inhibited macrophage apoptosis by MAPK‐NF‐κB pathway. A, MAPK family proteins and (B) NF‐κB p65 protein were determined by Western blot from cell lysates of 60 μmol/L 7‐KC–stimulated macrophages at different time‐points (0, 3, 6, 12, 18, 24 h). C, Western blot analysis of MAPK family proteins and (D) NF‐κB p65 protein from cell lysates of 7‐KC‐stimulated macrophages in the presence of either Rap (5 μmol/L) or CQ (25 μmol/L). Data were presented as mean ± SEM of at least three independent experiments. *P < .05 vs control, **P < .01 vs control, #*P* < .05 vs 7‐KC group, ##*P* < .01 vs 7‐KC group

To investigate the molecular mechanism involved in 7‐KC–induced macrophage apoptosis, cells were treated with 60 μmol/L 7‐KC at different time‐points. As shown in Figure 6A,B, phosphorylation of JNK, p38 and ERK1/2 was gradually increased by 7‐KC treatment. Phosphorylation of NF‐κB p65 revealed similar tendency. These results indicated that the activation of MAPK‐NF‐κB signalling pathway could be involved in 7‐KC–induced macrophage apoptosis. To determine the effects of autophagy on phosphorylation of MAPKs and NF‐κB activation, cells were treated with 7‐KC, in combination with Rap or CQ. As shown in Figure 6C,D, rapamycin inhibited 7‐KC–induced MAPK and NF‐κB activation. However, CQ significantly enhanced 7‐KC–induced MAPK and NF‐κB activation. These results suggested that activation of autophagy suppressed MAPK and NF‐κB activation.

## 4 DISCUSSION

In the current study, we found that autophagy could inhibit macrophage apoptosis and improve the stability of vulnerable plaques. Meanwhile, we also verified that activation of autophagy inhibited cell apoptosis, via improving macrophage mitochondrial dysfunction and inhibiting MAPK‐NF‐κB signalling pathway.

In vulnerable plaques, the necrotic core of plaque, which is most composed of apoptotic macrophages, is a pivotal characteristic of vulnerable plaques that causes major adverse cardiovascular events and acute clinical complications, such as cardiovascular death and acute myocardial infarction.^3^ Autophagy, a major intracellular degradation system, regulates the degradation of proteins and damaged organelles in the lysosome and plays important roles in the balance of cells’ apoptosis and proliferation.^8^ Razani et al^9^ reported that autophagy is defective in atherosclerosis which promotes atherosclerosis. Moreover, recent studies indicated that different stimuli suppressing autophagic mechanisms in the atherosclerotic microenvironment exacerbated atherosclerosis lesion progression in both animal experimental models^33^, ^34^ and in human carotid atherosclerotic tissue.^35^, ^36^ However, vulnerable plaque is different from atherosclerotic plaque. Vulnerable plaque is more prone to rapid plaque progression which is more likely to become clinically evident such as coronary syndrome and stroke.^37^ Therefore, stabilizing vulnerable plaques and reducing the incidence of adverse clinical events will be a main goal in the treatment of cardiovascular disease. In this study, we demonstrated that activation of autophagy by rapamycin could enhance the stability of vulnerable plaques by increasing the collagen content and decreasing the plaque necrotic core and macrophage apoptosis; nevertheless, autophagy inhibitor 3‐MA displayed the opposite effects (Figure S2 and Figures 1, 2). These results indicate that activation of autophagy has a protective effect on stabilizing vulnerable plaques. Furthermore, we explored the potential mechanism upon regulation of the stability of vulnerable plaques by autophagy.

Macrophage apoptosis promotes the development of necrotic core, inflammation and even plaque rupture in advanced atherosclerosis.^29^ 7‐ketocholesterol, one of the primary oxysterols in oxLDL, has been suggested as important inducers of apoptosis in many cell types, including macrophage.^30^ Herein, 7‐ketocholesterol (7‐KC) treatment of macrophages is used as a cell model for analysing cell death, apoptosis and autophagy. In our study, we also found that 7‐KC–induced macrophage apoptosis in a time‐ and dose‐dependent manner via activating the proteins of caspase‐9 and caspase‐3 (Figure S3).

During autophagy, LC3I is converted to LC3II through lipidation by a ubiquitin‐like system.^38^ The presence of LC3 in autophagosomes and the conversion of LC3I to the lower migrating form (LC3II) have been used as indicators of autophagy.^39^ Meanwhile, lysosomal degradation of autophagosomes leads to a decrease in SQSTM1 levels. Also, p62 combines with ubiquitinated proteins and binds to LC3II proteins to form a complex that is eventually degraded in the lysosome when autophagy occurs.^40^ Therefore, the simultaneous accumulation of LC3II and p62 is in fact a consequence of a blockade of the autophagic flux. In the present study, high concentration of 7‐KC alone obviously increased the ratio of LC3II/LC3I and the SQSTM1/p62 protein expression (Figure S4A), which indicated a blockage of cargo degradation. This further proved that proatherogenic stimulation (7‐KC) could impair autophagy flux, at least in part, through inhibiting the fusion of autophagosomes and lysosomes, which is consistent with recent reports.^41^ Rapamycin, the prototypical inhibitor of mTOR, as a strong autophagy inducer, has been shown to not only affect autophagosome formation,^42^ but also facilitates autophagic flux via regulating autophagosome‐lysosome fusion to promote the degradation of autophagy‐related proteins.^43^ 3‐MA influences autophagy via inhibiting its formation at an early stage, whereas CQ inhibits the fusion of autophagy with lysosome at a later stage.^42^ Similarly, we verified that cocultivation with rapamycin activated autophagy with degradation of SQSTM1 and LC3II proteins. In addition, cocultivation with 3‐MA or CQ impaired autophagy flux (Figure S4B,C).

Autophagy is of great significance for the homeostasis of macrophage,^44^ which may play protective role in macrophage apoptosis. Next, we would like to investigate whether autophagy reversed the role of 7‐KC on macrophage apoptosis. In the present study, our findings showed that activation of autophagy with rapamycin could suppress apoptosis induced by 7‐KC in RAW264.7 cells (Figure 3). In murine macrophages, we also verified that rapamycin had the same influence on regulating apoptosis induced by 7‐KC (Supplementary Figure S5). However, inhibition of autophagy with 3‐MA, CQ or Atg5 siRNA exacerbated cell apoptosis induced by 7‐KC (Figure 4). All these results are in consistent with previous studies that other types of cells (eg VSMCs, pancreatic β cells and neurons) are also more susceptible to injury‐mediated cell apoptosis under defective or inductive autophagy conditions.^45^, ^46^

Mitochondrial dysfunction is characterized by immoderate mitochondrial fission and reduction of mitochondrial membrane potential (MMP), which induces excessive ROS and release of cytochrome c, which can activate the mitochondrial apoptosis pathways.^32^ For the latter, cytochrome c is released to the cytoplasm for the high permeability of the mitochondrial outer membrane. Cytochrome c assembles procaspase‐9, activates caspase‐3 in succession and finally induces apoptosis.^47^, ^48^ Recent studies have indicated that an excess generation of mitochondrial ROS plays a crucial role in the pathogenesis of atherosclerosis.^24^, ^49^ Meanwhile, mitochondrial fission leads to mitochondrial fragmentation and cell apoptosis.^31^ In this study, 7‐KC treatment in macrophages induced the reduction mitochondrial membrane potential (MMP), significant ROS and cytochrome c release, as well as mitochondrial fragmentation and the impairment of mitochondrial ultrastructure. These findings suggest that 7‐KC treatment induces mitochondrial dysfunction, which subsequently induces cells apoptosis in macrophages. Also, pretreatment with rapamycin could effectively improve mitochondrial dysfunction. On the contrary, pretreatment of cells with autophagy inhibitors could aggravate mitochondrial dysfunction (Figure 5). Taken together, we can conclude that the effect of anti‐apoptosis by autophagy induction was associated with modulating mitochondrial homeostasis.

Finally, we explored the signalling pathway involved in 7‐KC–induced macrophage apoptosis by autophagy. The MAPKs consist of a series of highly conserved serine/threonine protein kinases that are three main classes of MAPK family in mammals, ERK1/2, JNK and p38.^50^, ^51^ MAPK pathway can regulate many cellular processes, cell proliferation, apoptosis and differentiation,^52^ which can be modulated by ROS‐dependent redox cycling.^53^, ^54^, ^55^ Therefore, we speculated that MAPK pathway might be involved in the regulation of 7‐KC–induced apoptosis by autophagy. In our study, 7‐KC significantly induced activation of MAPK pathway. Then, we observed that rapamycin obviously inhibited 7‐KC–induced phosphorylation of ERK1/2, JNK and p38. However, CQ aggravated activation of MAPK pathway (Figure 6).

It has been reported that phosphorylation of MAPKs could induce activation of NF‐κB, leading to cell apoptosis.^56^ NF‐kB, one of the inducible transcription factors, is controlled by a family of IκBα inhibitory proteins, which is retained in the cytoplasm in resting cells. Upon stimulation, IκBα proteins are phosphorylated, undergo ubiquitination and degradation and allow NF‐κB translocation to the nucleus, where it influences the expression of numerous genes including those involved in inflammation, cell cycle and apoptosis.^57^, ^58^ In other studies, NF‐κB activates transcription of multiple genes involved in the regulation of cell survival or apoptosis in a cell type‐ and stimulus‐dependent manner.^59^, ^60^ It seems that NF‐κB activity plays an important role in the regulation of cell apoptosis. In the present study, our results suggested that 7‐KC–induced NF‐κB activity in a time‐dependent manner. Then, we found that administration with rapamycin could alleviate NF‐κB activity. Nevertheless, CQ increased 7‐KC–induced phosphorylation of NF‐κB (Figure 6). These data suggest that autophagy can inhibit macrophage apoptosis via regulating the activation of MAPK‐NF‐κB signalling pathway.

**Figure 7:**
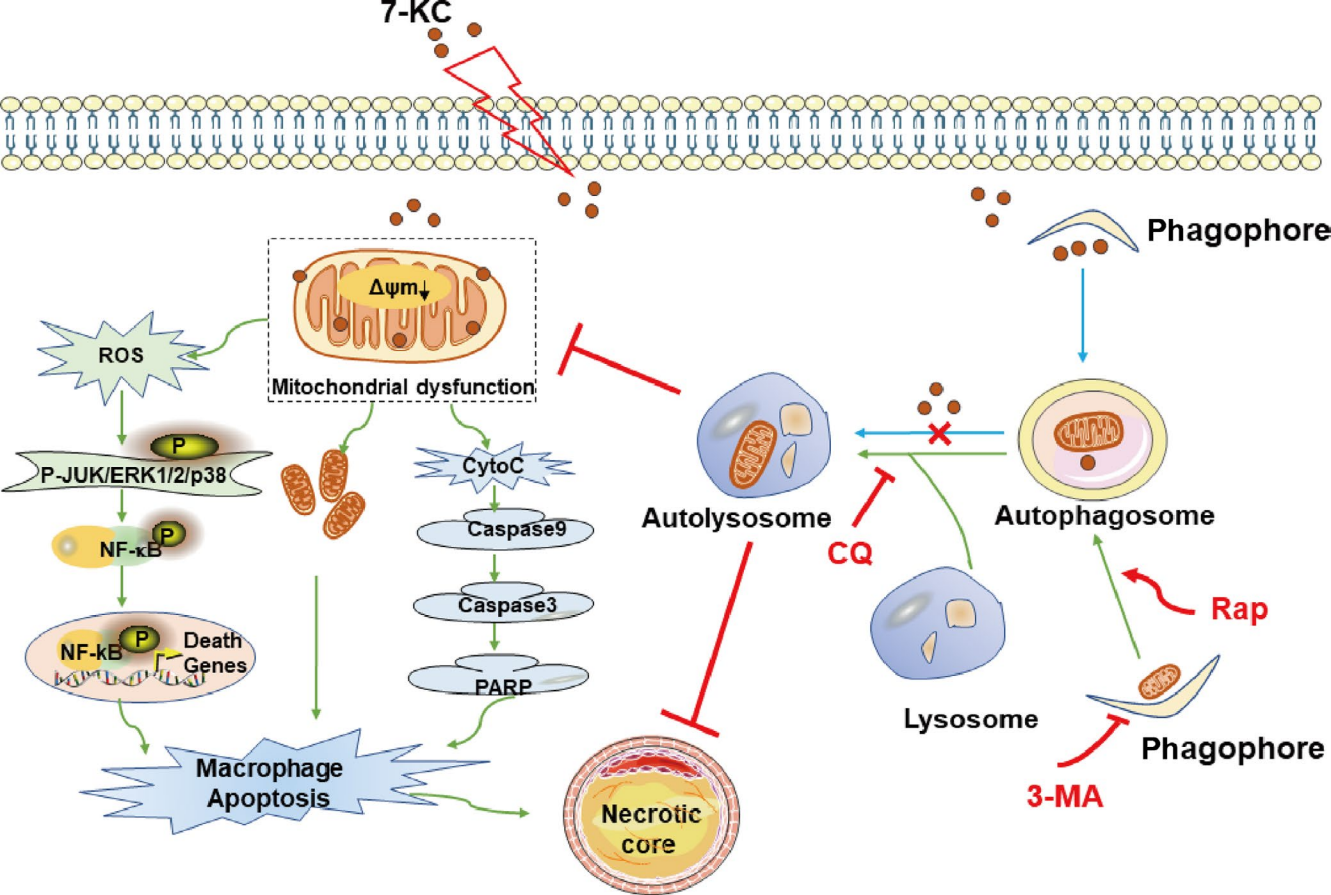
Graphic summary of the effects of autophagy on necrotic core formation mediated by mitochondria‐mediated macrophage apoptosis. As shown in the graphic summary, 7‐KC impairs autophagy flux through mechanisms that might involve in lysosomal leakage or cholesterol crystal overloading. Meanwhile, 7‐KC leads to mitochondrial dysfunction, indicated by down‐regulated Δψm, excessive ROS generation and cytochrome c release, along with increased mitochondrial fragmentation. Increasing of ROS levels bring about activity of MAPK‐NF‐κB signalling pathway. Meanwhile, cytochrome c release activates caspase cascade pathway. All of these eventually cause macrophage apoptosis which promotes necrotic core formation in vulnerable plaques. Moreover, the activation of autophagy will lead to autophagosome and autolysosome formation, which inhibits mitochondrial dysfunction. Thus, autophagy protects against mitochondria‐mediated macrophage apoptosis and inhibits necrotic core formation in vulnerable plaques

In summary, our study demonstrated that autophagy alleviated vulnerable plaques instability with reducing incidence of intraplaque haemorrhage and plaque rupture. Also, autophagy inhibited necrotic core formation by suppressing intraplaque macrophage apoptosis. Moreover, macrophages autophagy could effectively ameliorate mitochondrial dysfunction and cell apoptosis, suggesting that autophagy may regulate mitochondria‐mediated apoptosis for stabilizing vulnerable plaques. Meanwhile, autophagy could have cytoprotection in macrophage via inhibition of MAPK‐NF‐κB signalling pathway (Figure 7). Macrophage autophagy probably offers a novel therapeutic target for vulnerable plaques. However, there are still some complicated molecular mechanisms need to be further explored such as the way that autophagy regulates endoplasmic reticulum stress. Additionally, these findings need to be verified by further studies in autophagy gene knockout mice experiments.

## CONFLICT OF INTEREST

Nothing to declare.

## AUTHOR CONTRIBUTIONS

QS and JP originally designed the research. QX, XC and BC performed experiments. All the authors analysed data. QS and QX wrote the manuscript. JP and QX edited the manuscript. All authors approved the submitted and final versions.

## Data Availability

The data that support the findings of this study are available from the corresponding author upon reasonable request.
