# Supplementary figures and images for: Macrophage autophagy regulates mitochondria‐mediated apoptosis and inhibits necrotic core formation in vulnerable plaques
Source: J Cell Mol Med. 2019 Oct 29;24(1):260–75. doi: 10.1111/jcmm.14715 (PMC6933382; doi:10.1111/jcmm.14715)

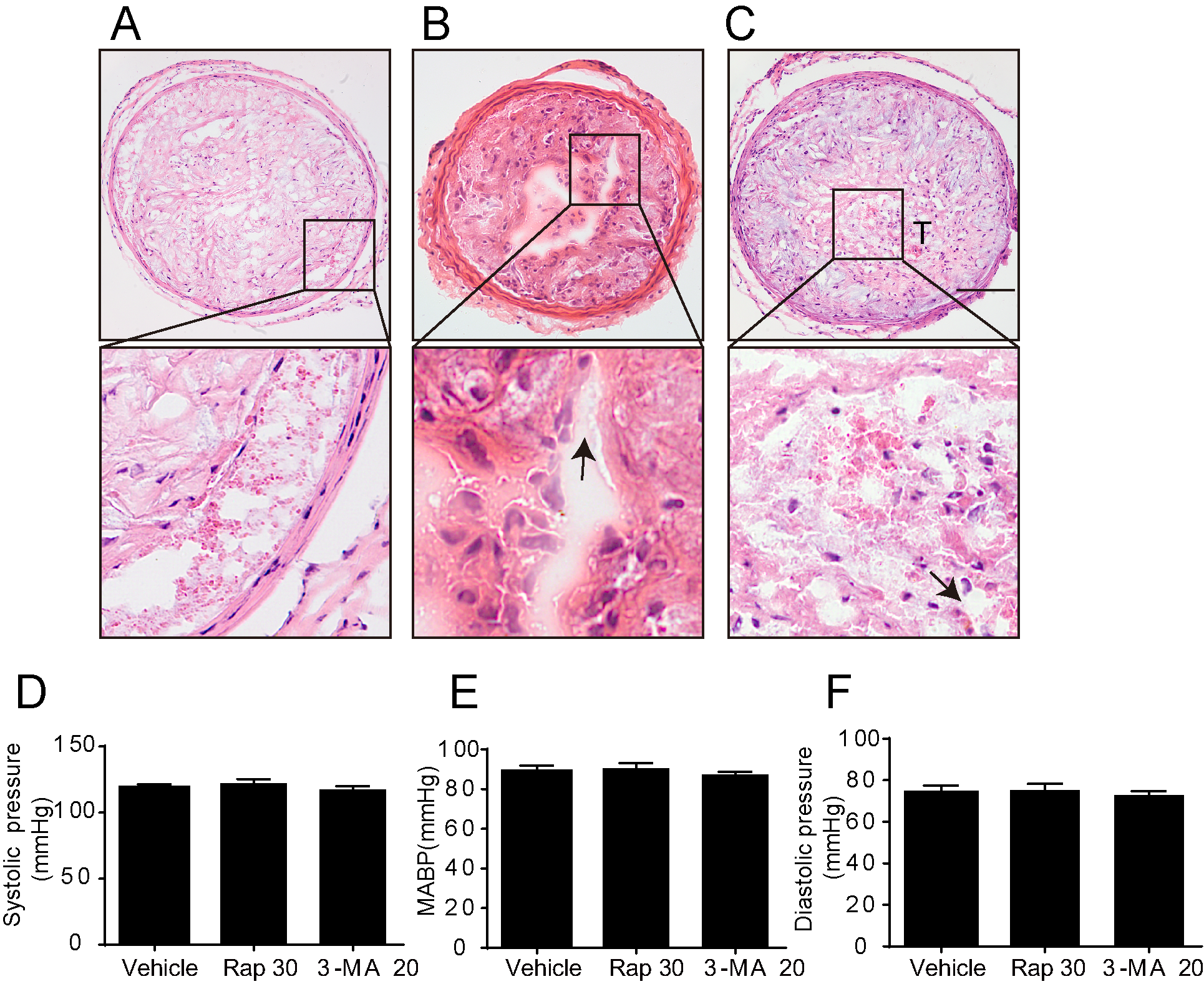

Supplement: Supplementary file 1 [file JCMM-24-260-s001.tif]

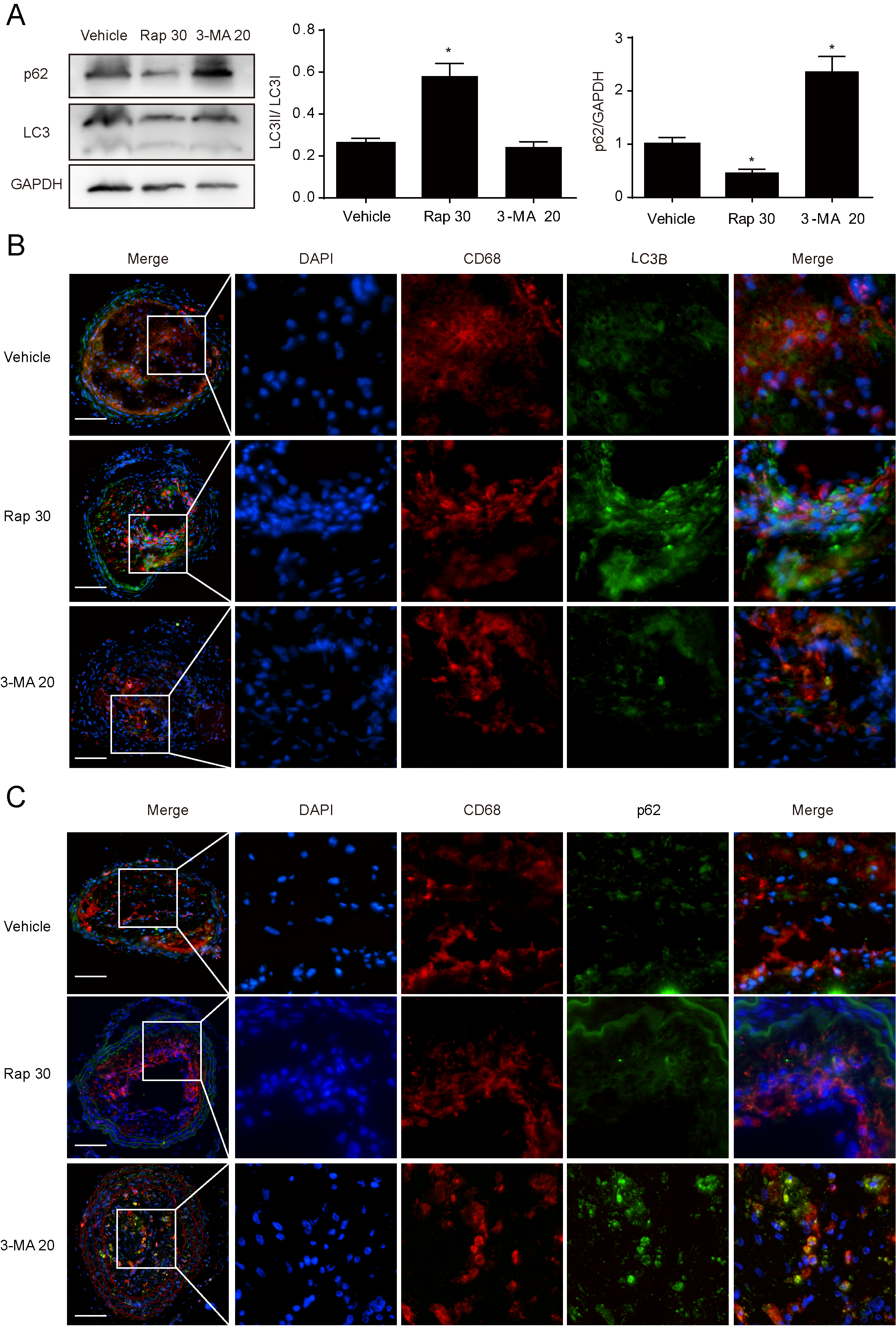

Supplement: Supplementary file 2 [file JCMM-24-260-s002.tif]

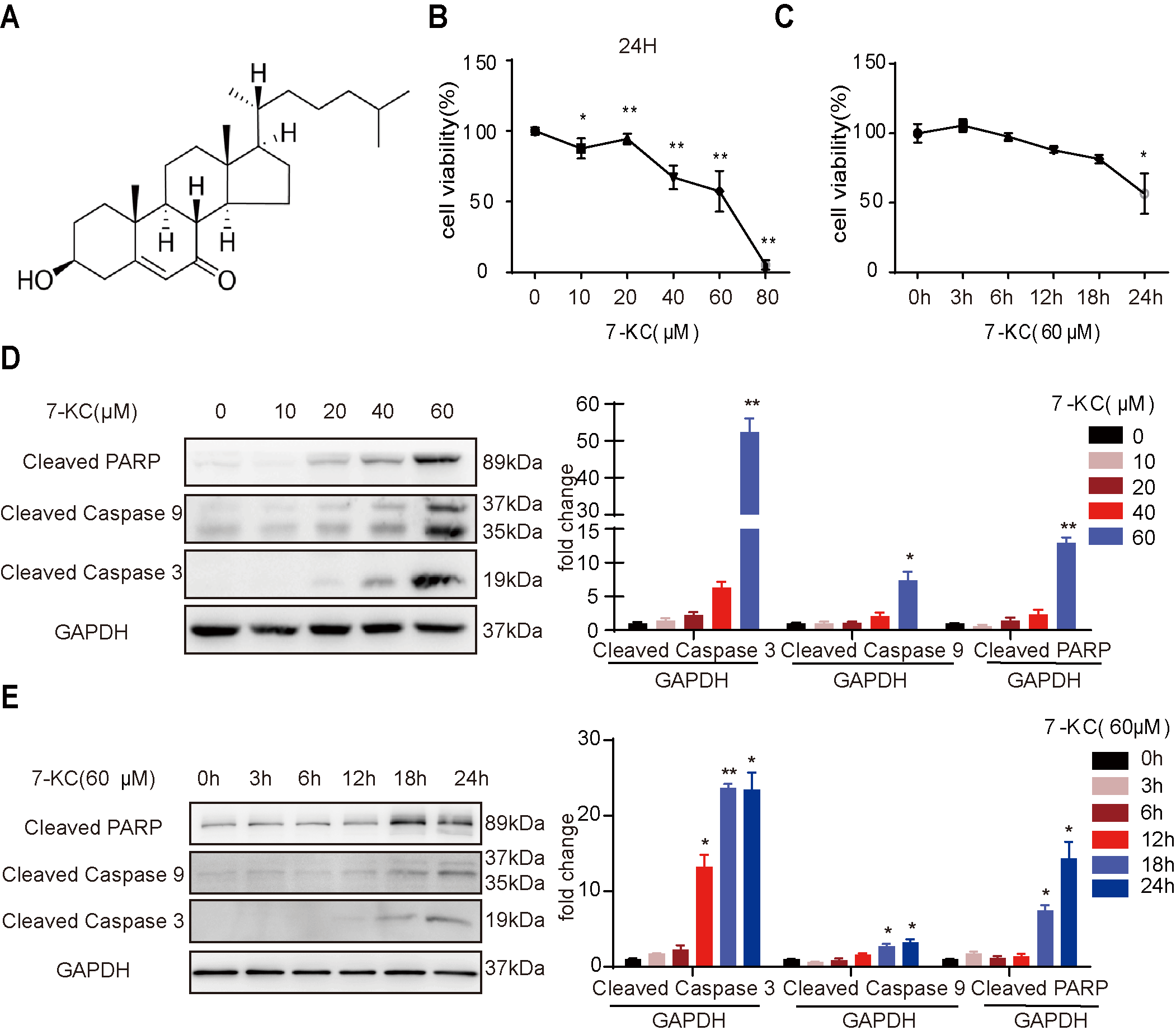

Supplement: Supplementary file 3 [file JCMM-24-260-s003.tif]

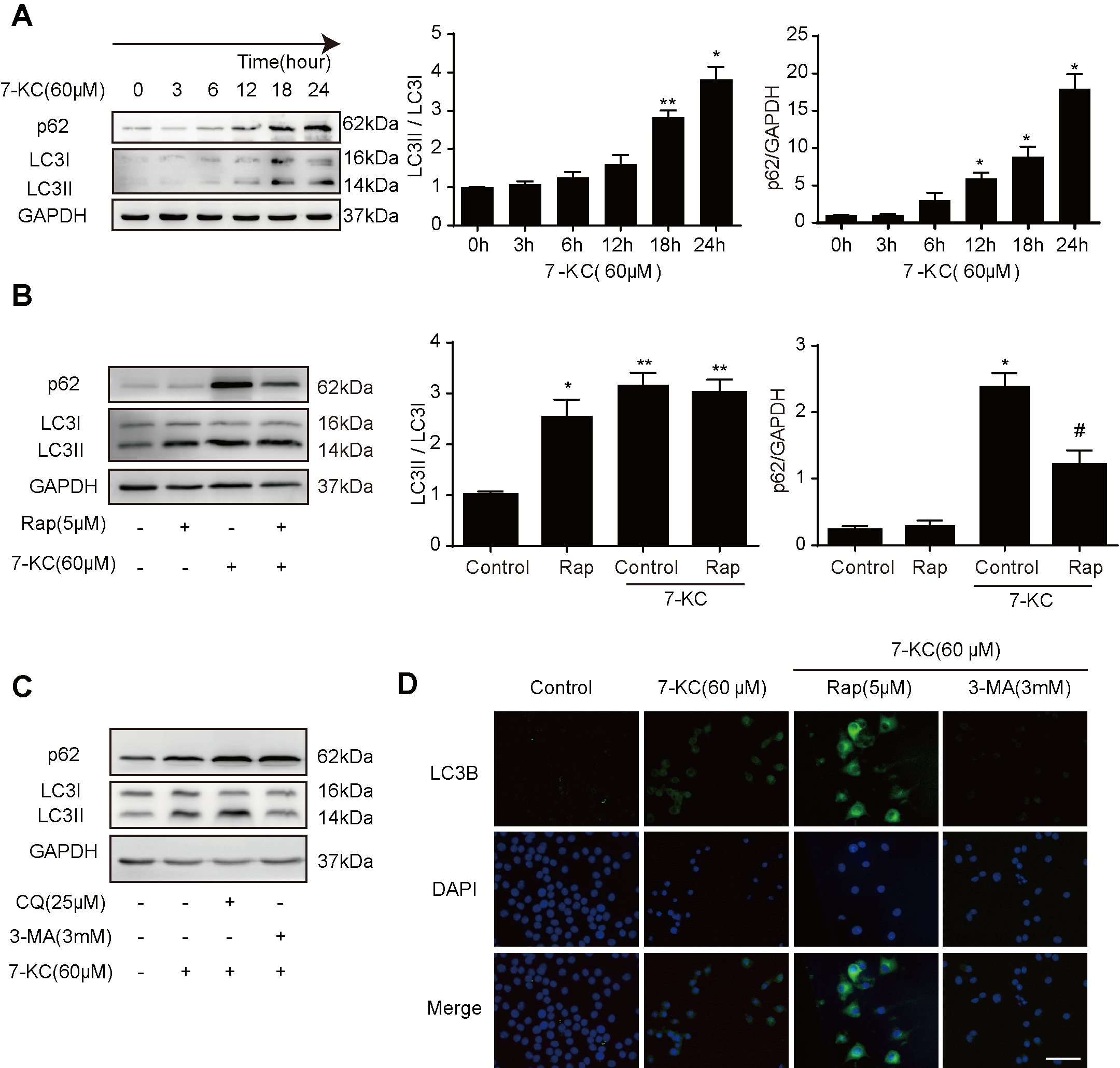

Supplement: Supplementary file 4 [file JCMM-24-260-s004.tif]

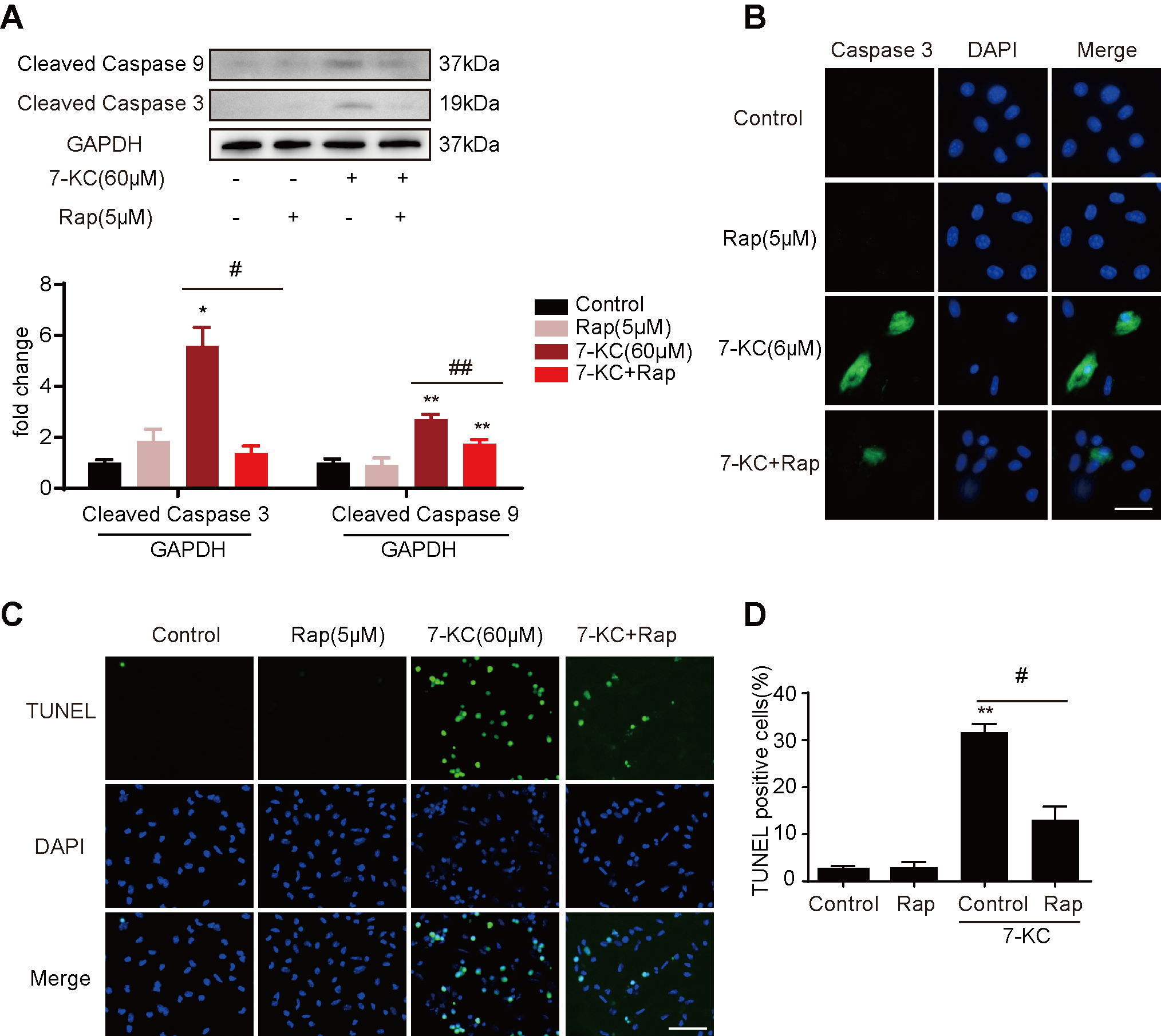

Supplement: Supplementary file 5 [file JCMM-24-260-s005.tif]
