# Supplementary material for: Macrophage autophagy regulates mitochondria‐mediated apoptosis and inhibits necrotic core formation in vulnerable plaques
Source: J Cell Mol Med. 2019 Oct 29;24(1):260–75. doi: 10.1111/jcmm.14715 (PMC6933382; doi:10.1111/jcmm.14715)
Supplement: Supplementary file 6 [file JCMM-24-260-s006.doc]

**Macrophage autophagy regulates mitochondria-mediated apoptosis and inhibits necrotic core formation in vulnerable plaques**

**Supplement Material**

**Materials and Methods：**

**1.Materials:**

Rabbit polyclonal anti- Caspase-3, anti-LC3, anti-SQSTM1/p62, anti-GAPDH and Rabbit monoclonal anti- Caspase-9, anti-PARP, anti- Cytochrome C, anti- COX Ⅳ, anti-phospho-ERK1/2, anti-ERK1/2, anti-phosphor-SAPK/JNK, anti- JNK, anti-phospho-p38 MAPK were obtained from Cell Signaling Technology Inc. (Danvers, MA, USA). Rabbit monoclonal anti-LC3B, anti-SQSTM1/p62 were purchased from Abcam (Cambridge, MA, USA).7-KC(7-ketocholesterol), 3-MA (3-Methyladenine), MTT [3-(4, 5)-dimethylthiahiazo (-2-y1)-2, 5-diphenyl-tetrazolium bromide] were obtained from Sigma (St. Louis, MO, USA). Rap (rapamycin) and CQ (Chloroquine) were purchased from MCE (Monmouth, NJ, USA). All the other chemicals were acquired from commercial sources.

**2. MRI**

Magnetic resonance imaging (MRI) was performed at weeks 4 and 8 after combined surgery. All scanning was carried out with a 7.0-T small animal, Superconducting magnet and BioSpec spectrometer (BioSpec 70/20 USR: Bruker, Bruker Biospin, Ettlingen, Germany). All mice were induced and maintained under isoflurane anesthesia (1.5% to 2%) in medical-grade and monitored simultaneously via a sensor positioned on the abdomen for respiration rate (30-50 breath/min). All animals were placed in the prone position. MRI images were acquired using spoiled gradient recalled echo T2 Star imaging (FLASH_T2star) and black-blood T2 to proton density (PD)-weighted multi-echo multi-spin (MEMS) Sequence (MEMS_PD_T2). Imaging parameters were as follows:FLASH_T2star: repetition time (TR) = 950 ms, echo time (TE) = 5ms, FOV = 2.5 cm, matrix size = 256×256, and in-plane resolution = 98μm × 98μm×500μm, slice thickness = 0.5 mm, slices=20 ; MEMS_PD_T2: TR 2468.970ms, TE = 8.667ms, FOV = 2.5 cm, matrix size = 256×256, and in-plane resolution = 98μm×8μm×500μm, slice thickness = 0.5 mm, slices=20. The imaging acquisition time was 45 minutes per animal. The obtained images were analyzed using RadiAnt DICOM Viewer (Poznan, Poland).

**3. Western blot Analysis**

Cells were homogenized in a lysis buffer with PMSF (Roche, USA) for 30 min on the ice and the supernatant fluid was collected. Proteins were separated by 6%-12.5% SDS-polyacrylamide gel electrophoresis and transferred to polyvinylidene difluoride (PVDF) membrane (Millipore, Bedford, MA) by electrotransfer (250 mA for 90 min). After blocking with 5% non-fat dry milk in TBST for 1h at room temperature, the membranes were washed three times with TBST each for 5 min, incubated with primary antibodies overnight at 4°C. Then, the membranes were rinsed three times with TBST and incubated with horseradish peroxidase-conjugated anti-mouse or anti-rabbit IgG for 1h at room temperature. Simultaneously, GAPDH acted as the control. Subsequently, the chemiluminescent bands were recorded using LAS-4000 mini system (Fujifilm, Japan).

1. **Flow cytometric analysis of apoptosis by annexin V/PI staining**

RAW264.7 cells were collected and washed twice with cold PBS (1000 rpm, 5min). Approximately 5 × 10^5^ cells were resuspended in 195μL binding buffer and then incubated with 5μL annexin V-FITC with 10μL PI (Beyotime Institute of Biotechnology) at room temperature for 20min. The samples were detected by flow cytometer (BD, USA).

1. **Real-time quantitative PCR**

The cells (2×10^5^ cells/well in 24-well plates) were transfected with control siRNA(siCtrl) or Atg5 siRNAs(siAtg5) at the indicated times. The total RNA was isolated with the Trizol reagent (Life Technologies). The cDNA synthesis was carried out with 500 ng of total RNA that was primed with random (dT). Quantitative real-time PCR was performed and specific primers were designed as follows:

Atg5: forward primer, 5’-CCCCAGCCAACAGATTGA A-3’ and

reverse primer, 5’-GCCTCCACTGAACTTGACTGt-3’;

GAPDH: forward primer, 5’- ACTCCACTCACGGCAAATTCA-3’ and

reverse primer, 5’-GGCCTCACCCCATTTGATG-3’

Real-time quantitative PCR was performed using SYBR Green dye and the LightCycler 480 Real-Time PCR System (Roche Applied Science, Indianapolis, IN). The resulting values were normalized to GAPDH expression.

**6. Macrophage transfection with siRNA**

Small interfering RNAs (siRNA) against Atg5 and control siRNA were synthesized by GenePharma (Shanghai, China). The following siRNA sequences were used:

（1）ATG5siRNA-1:5′-GACGUUGGUAACUGACAAATT tt-3′

5′- UUUGUCAGUUACCAACGUCTT tt-3′;

（2）ATG5 siRNA-2:5′- GCAUUAUCCAAUUGGUUUATT tt-3′

5′- UAAACCAAUUGGAUAAUGCTT tt-3′;

Cells were seeded in 6-well plates and incubated overnight. After that, cells were transfected with siRNA targeting Atg5 (100 nM) or scrambled control siRNA (100 nM) using RNAiMAX (Invitrogen) according to the manufacturer's instructions. After transfection for 24h, the mRNA and protein expression of the cells were detected, respectively.

**7. Immunofluorescence Staining**

The macrophages were washed thrice with PBS, fixed with 4% paraformaldehyde solution for 15 min and permeabilized using 0.1% Triton X-100 for 10 min at room temperature. After washing with PBS three times, the cells were blocked with 5% BSA for 1h at room temperature and incubated with anti-LC3 and anti-cleaved caspase 3 antibody (both in 1:200, Abcam, Rabbit IgG) overnight at 4℃. After rinsed with PBS three times, the cells were incubated for 1h with goat anti-rabbit IgG/Alexa Fluor 488 antibody (1:200, Invitrogen). Cells were then mounted with DAPI (Life Technologies, Grand Island, NY, United States) for nuclei staining. Finally, images were acquired on a fluorescence microscope (Leica DM3000B, Germany).

**8. Terminal deoxynucleotidyl transferase dUTP nick end labeling (TUNEL) assay**

Cell apoptosis was determined by In Situ Cell Death Detection K（Roche, Mannheim, Germany). Tissue sections or macrophages were fixed with 4% paraformaldehyde for 20 min and permeabilized with 0.1% Triton X-100 in PBS for 1h at room temperature, and then, rinsed with PBS three times, incubated in permeabilization solution (0.1% Triton X-100 containing 0.1% sodium citrate) for 2 min on ice. Sections or cells were then incubated with TUNEL reaction mixture for 60 min at room temperature. Afterwards, they were incubated with DAPI for 15min. Finally, sections or cells were analyzed with a fluorescence microscope (Leica DM3000B, Germany). The percentage of TUNEL positive (green) cells was analyzing using an ImageJ software.

**9. Transmission electron microscopy (TEM)**

The ultrastructure of mitochondrial in RAW264.7 cells was examined via TEM. Briefly, macrophage cells were cocultured with Rap (rapamycin;5μM) for 1 h and subsequently incubated with 60μM 7-KC for 24h. Then, cells were collected and fixed with 2% glutaraldehyde for 2 h and treated with 1% osmium tetroxide for 2 h. The samples were then dehydrated and embedded in Epon-Araldite resin. The ultrastructure of mitochondrial in cells are visualized with a Tecnai G2 12 transmission electron microscope (FEI Company, Holland).

**10. Detection of intracellular ROS**

Intracellular ROS levels were detected by analyzing the intensity of the DCF signal by flow cytometry analysis. The treated RAW264.7 cells (1×10^6^cells/ml) were incubated with 10μmol/L DCFH-DA diluted in DMEM medium at 37°C for 50 min. Cells were then washed twice with PBS. Immediately after washing, the cells were measured under a flow cytometer (BD, USA).

**11. Measurement of Mitochondrial Membrane Potential (ΔΨ _m_)**

To detect the mitochondrial membrane potential (ΔΨ _m_) of RAW264.7 cells, JC-1 (5,5',6,6'-tetrachloro-1,1',3,3'- tetra-ethylbenzimidazolylcarbocyanine iodide) (Beyotime, Jiangsu, China), mitochondrial membrane potential sensitive probe was used. Briefly, the cells were seeded in 24-well plates at a density of 1×10^5^ cells per well. After 24 hours of treatment, the cells were stained with 5 μM JC-1 for 20 min at 37°C. Meanwhile, cells treated with 10μM CCCP for 20 min before JC-1 staining were used as a positive control. Then, cells were washed with ice-cold PBS twice, and immediately analyzed with a fluorescence microscope (Leica DM3000B, Germany). For analyzing of JC-1 monomers, the wavelengths of excitation and emission were 490 nm and 530 nm, and for JC-1 aggregates, the wavelengths were 525 nm excitation and 590 nm emission. The change from green-to-red fluorescence ratio was served as a measure of mitochondrial membrane potential.

**12. Extraction of mouse peritoneal macrophages**

Male C57BL/6 mice (8-12wk age) were purchased from Shanghai Jiesijie laboratory Animal Co, Ltd and administered intraperitoneally with 2 ml of 3% thioglycolate broth medium for 72 h. Then, the mice were euthanized and submerged in 75% ethanol for 4 min, and the peritoneal macrophages were harvested via peritoneal lavage with 15ml cold phosphate-buffered saline (PBS). The cells were cultured in RPMI 1640 medium supplemented with 10% FBS, 100 μg/mL streptomycin and 60 units/mL penicillin at 37℃ in 5% CO2. Six hours later, nonadherent cells were washed off with warm PBS, and adherent peritoneal macrophages were used for subsequent experiments.

**Supplementary Figure Legends**

**Supplementary Figure S1. Typical characteristic of vulnerable plaques and changes of blood pressure**

Representative images of carotid artery cross sections stained with hematoxylin and eosin (H&E) from ApoE^-/-^ mice. A. Intraplaque hemorrhage was observed in lesions with intact fibrous cap and necrotic core containing red blood cells and fibrin; B-C, Plaque rupture (the black arrows) with thrombus (T) were observed in lesions, scale bar = 100 μm. D-F. systolic pressure, diastolic pressure and mean arterial blood pressure (MABP) were measured at 8 weeks after combined surgery(n=15)

**Supplementary Figure S2.** Rapamycin and 3-MA modulated macrophage autophagy in vulnerable plaques in ApoE^−/−^ mice

(A)The protein levels of LC3 and p62 (SQSTM1/p62) were determined by Western blot in whole-aorta lysates from vehicle, rapamycin and 3-MA- treated mice. (B-C) Representative images of carotid artery cross sections were stained with CD68 antibodies to label macrophages, and LC3B or p62 antibodies to identify macrophage autophagy, scale bar = 100 μm. Data were presented as mean ± SEM of at least three independent experiments. *P < 0.05 compared with Vehicle group, **P < 0.01 compared with Vehicle group. Vehicle, mice were administrated with saline solution alone. Rap 30, Rapamycin at 30 mg/kg/day; 3-MA 20, 3-Methyladenine at 20 mg/kg/day.

**Supplementary Figure S3. 7-KC treatment caused apoptosis in RAW264.7 cells**

(A)Chemical structure of 7-ketocholesterol(7-KC). (B) RAW264.7 macrophages were exposed to 7-KC at the different concentrations (0, 10, 20, 40, 60, 80μM) for 24h. Cell viability was measured by MTT assay. (C)Cells were incubated with 60μM 7-KC at different time intervals (0, 3, 6, 12, 18, 24h), then cell viability was measured by MTT assay. (D) Cells were incubated with 7-KC at the concentrations indicated (0, 10, 20, 40, 60μM) for 24h. The protein levels of cleaved caspase 3, cleaved caspase 9 and cleaved PARP were evaluated by Western blot. (E)Cells were incubated with 60μM 7-KC at different time intervals (0, 3, 6, 12, 18, 24h). The protein levels of cleaved caspase 3, cleaved caspase 9 and cleaved PARP were determined by Western blot. Data were presented as mean ± SEM of at least three independent experiments. *P < 0.05 compared with control group, **P < 0.01 compared with control group.

**Supplementary Figure S4. Autophagy flux was blocked during 7-KC-induced macrophages apoptosis**

(A) RAW264.7 cells were incubated with 60μM 7-KC at different time intervals (0, 3, 6, 12, 18, 24h). The protein levels of LC3 and p62 (SQSTM1/p62) were determined by Western blot. (B) Cells were pretreated with Rap (rapamycin, 5μM) or control for 1 h, followed by treatment with 7-KC (60μM) for additional 24h. The protein levels of LC3 and p62 (SQSTM1/p62) were also determined by Western blot. (C) Cells were treated with 7-KC (60μM) in the absence or presence of 3-MA (3-methyladenine, 3 mM) or CQ (chloroquine, 25μM) for 24h. The protein levels of LC3 and p62 (SQSTM1/p62) were also determined by Western blot. (D) Cells were treated with 7-KC (60μM) in the absence or presence of Rap (5μM) or 3-MA (3 mM) for 24h. Then cells were stained by LC3B (green) and DAPI (blue) and analyzed by fluorescence microscopy, scale bar = 50 μm. Data were presented as mean ± SEM of at least three independent experiments. *p<0.05 *vs.* control, ** p<0.01 *vs.* control, #p<0.05 *vs.* 7-KC group.

**Supplementary Figure S5. Rapamycin inhibited cells apoptosis induced by 7-KC in peritoneal macrophages**

Peritoneal macrophages were treated with Rap (5μM) for 60 min, and then exposed to 7-KC (60μM) for additional 24h. (A) Western blot analysis of cleaved caspase 3 and cleaved caspase 9 expression in the whole cells. (B) Cells were stained by cleaved caspase3 (green) and DAPI (blue) and analyzed by fluorescence microscopy, scale bar =25 μm. (C-D) Representative images of TUNEL staining of macrophages showed the apoptotic cells (apoptotic cells stained in green and nucleus stained in blue with DAPI). The number of TUNEL-positive cells was measured and quantitated, scale bar = 65 μm. Data were presented as mean ± SEM of at least three independent experiments. *p<0.05 *vs.* control, ** p<0.01 *vs*. control, #p<0.05 *vs*. 7-KC group.
